# Serum Soluble CD14 Is a Potential Prognostic Indicator of Recurrence of Human Breast Invasive Ductal Carcinoma with Her2-Enriched Subtype

**DOI:** 10.1371/journal.pone.0075366

**Published:** 2013-09-25

**Authors:** Weifeng He, Yifan Tong, Ying Wang, Jingjing Liu, Gaoxing Luo, Jun Wu, Jin Zhang

**Affiliations:** 1 Chongqing Key Laboratory for Disease Proteomics, State Key Laboratory of Trauma, Burns and Combined Injury; Institute of Burn Research, Southwest Hospital, Third Military Medical University, Chongqing, PR China; 2 3rd Department of Breast Cancer, China Tianjin Breast Cancer Prevention, Treatment and Research Center, Tianjin Medical University, Cancer Institute and Hospital, Tianjin, PR China; The University of Hong Kong, China

## Abstract

In clinical practice, breast cancers with lymph node positive, ER/PR-negative and overexpressed human epidermal growth factor receptor 2 (LN+ER/PR-Her2+) have high risk of recurrence, but the effective biomarkers of prognostic for this type tumor are still lacking. Since breast cancers with LN+ER/PR-Her2+ is at higher risk of recurrence than those with LN-ER/PR+Her2-. The differential proteins between those two groups could be related to the risk of recurrence. Herein, we report that serum soluble CD14 (sCD14) was revealed as the stable differential protein between LN+ER/PR-Her2+ (n=50) and LN-ER/PR+Her2- (n=50) breast cancer patients by proteomics analysis. To validate sCD14 as a biomarker for predicting recurrence of breast cancer, 90 breast cancer patients with LN+ER/PR-Her2+ and 93 patients with LN-ER/PR+Her2- were recruited. The patients with higher level of serum sCD14 at primary surgery showed to be at significantly lower risk of relapse in 3 years follow-up than those with lower level of serum sCD14 at primary surgery. The levels of serum sCD14 at primary surgery were significantly correlated to the risk of 3-year recurrence of LN+ER/PR-Her2+ breast cancer and the corresponding AUC of the ROC curve was 0.833 (95% CI, and 0.742 to 0.920). Therefore, we surmise that serum sCD14 could be a potential biomarker for predicting the prognosis of breast invasive ductal carcinoma with LN+ER/PR-Her2+.

## Introduction

Breast cancer is the leading cause of cancer death in females worldwide [1] and the identification of its prognostic biomarkers could be valuable for improving the clinical management. Breast cancer with overexpressed human epidermal growth factor receptor 2 (Her2) is characterized by aggressive progress and shortened survival [2,3]. Practically, the receptors of breast cancer cells such as Her2, estrogen receptor (ER) and progesterone receptor (PR) have been clinically used as markers reflecting the prognosis of breast cancer [4,5], i.e. The patients with lymph node-positive tumors, ER/PR-negative and HER-2 over-expressing (LN+ER/PR-Her2+) are at high risk for cancer recurrence, and patients with lymph node negative, ER/PR-positive and HER-2 negative tumors (LN-ER/PR+Her2-) are at low risk for cancer recurrence [6]. However, the effective prognostic biomarkers for the two types of breast cancers are still lacking. It’s reasonable to assume that the differential serum proteins between those two groups might be correlated to the risk of breast cancer relapse. Thus, we comparatively studied the serum protein profile of breast invasive ductal carcinoma with a lower risk of recurrence (LN-ER/PR+Her2-; n=50) and those with a higher risk of recurrence (LN+ER/PR-Her2+; n=50) by proteomics, and then proposed that serum sCD14 is a potential biomarker for predicting recurrence of LN+ER/PR-Her2+ status breast cancer.

## Materials and Methods

### Ethics Statement

The study has been approved by the Ethics Committee of the Tianjin Medical University Cancer Institute and Hospital, Tianjin, and adhered to the tenets of the Declaration of Helsinki. In addition, written informed consent was obtained from the patients or their next of kin in this study.

### Study Participants

All breast invasive ductal carcinoma patients were the first diagnosis and without any pre-clinical treatment. A total of 183 histologically proved breast invasive ductal carcinoma cases were selected from April 2007 to January 2009, of which 93 cases were LN-ER/PR+Her2- status, another 90 cases were LN+ER/PR-Her2+ status. HER-2, ER and PR expression content was determined by using the Immunoenzymatic Staining. All LN-ER/PR+Her2- status and LN+ER/PR-Her2+ status breast cancer patients were taken with comparable therapy, respectively. The clinic pathologic characteristics of this cohort are summarized in Table 1.

**Table 1:** Patient demographics and clinical parameters.

	**LN-ER/PR+Her2- status**		**LN+ER/PR-Her2+ status**	
	**Relapse**	**No relapse**	**Relapse**	**No relapse**
Number of patients	12	81	39	51
Age in year	53.25±14.27	51.07±10.06	47.77±9.10	46.41±8.35
Time to relapse (months)	23.83±8.29	-	17.59±10.38	-
Pre-/post-menopausal	5/7	45/36	17/22	28/23
Tumor size (mm)	1.90±0.67	2.34±1.16	3.92±1.82	3.2±1.47
Family carcinoma history +/-	6/6	21/60	25/14	15/36
Stage I/IIa/IIb/III	8/2/2/0	46/29/6/0	6/14/9/10	17/23/7/4
Her2+/++/+++	-	-	14/20/5	23/19/9
ER content ≤30%/31%-60%/>60%	3/4/5	12/34/35	-	-
PR content ≤30%/31%-60%/>60%	4/0/8	8/30/43	-	-
PCNA content - / ≤30%/31%-60%/>60%	1/1/2/8	7/7/27/40	0/3/23/13	6/7/23/15
P53 content - / ≤30%/31%-60%/>60%	8/3/1/0	59/12/2/1	15/0/7/17	25/1/12/13
Lymph node <4/4-9/>9	-	-	16/13/10	34/12/5
Radiation/no radiation	12/0	81/0	39/0	51/0
Adjuvant therapy: hormonal (tamoxifen)	12/0	81/0	0/39	0/51

Patients were followed up quarterly for the 3 years. Chest x-ray was performed every six months for the first five years and then every year. Bone and liver scans and mammography were performed every year. If any symptoms or signs suggestive of a potential recurrence were detected or reported by the patients, focused investigations were carried out.

### Serum Sample Preparation

Blood samples were collected at the time of primary surgery. Briefly, vein blood (5ml) was collected in a serum separator tube, clotted for 30 minutes followed by centrifugation. Serum was then freshly frozen at -80°C. On one hand, for the proteomics analysis, 10µl serum was taken from each of the 50 patients of breast cancer with LN-ER/PR+Her2- status and 50 patients with LN+ER/PR-Her2+ status to build the LN-ER/PR+Her2- status and the LN+ER/PR-Her2+ status pools, respectively. On the other hand, the rest of the serum samples were kept in -80°C for ELISA examination after preliminary proteomics screening. The serum samples were centrifuged at 10,000 × g for 30 min at 4°C to remove any cellular debris. The targeted high-abundance proteins (i.e. albumin, IgG, antitrypsin, IgA, transferrin, and haptoglobin) in the samples were depleted by using an immuno-affinity column (Agilent Technologies, Palo Alto, CA, USA). The amount of protein after depletion of high-abundance proteins was measured by Coomassie Protein Assay Kit (Pierce, Rockford, IL, USA).

### On-Line 2D LC ESI-MS/MS analysis [7]

200 µg high-abundance-protein-depleted serum protein mixtures were digested in-solution as described above. After desalted by reverse phase C18 StageTips, the protein digest (10 µl) was injected to a strong cation exchange (SCX) column (300 µm i.d. x 5 cm；Agilent Technologies, Waldbronn, Germany) and eluted with ten salt plug injections (5 mM-500 mM NaCl). Eleven fractions obtained from ten salt plug injections were then introduced into the HPLC-Chip/XCT Ultra Trap. The first four fractions were separated with two hour-long gradients (2% B to 40% B in 110 min and 40% B to 95% B in 10 minutes; where solvent A is 2% acetonitrile, 0.1% formic acid, and solvent B is 0.1% formic acid, 90% acetonitrile) and the remaining fractions with one hour-long gradient. 

### HPLC-Chip/MS Analysis [7]

The tryptic digested and desalted protein samples were analyzed with a HPLC-CHIP-MS/MS system consisting of a nano pump (G2226A, Agilent) with 4-channel micro-vacuum degasser (G1379B, Agilent), a microfluidic HPLC-Chip Cube interfaced to a XCT Ultra ion trap mass spectrometer (all Agilent Technologies). Peptides were injected on the enrichment column via an autosampler. The mobile phase consisted of solvents A (water with 0.1% formic acid) and B (90% ACN, 10% water with 0.1% formic acid). The column was eluted with a gradient from 3% B to 45% B in 90 min, followed by a steep gradient to 80% B in 10 min. The total analysis time was 110 min, and the flow rate was fixed at 0.3 µL/min. 

Data-dependent MS acquisition was performed on the Agilent LC/MSD Trap XCT with the following MS conditions: 4 L/min, 300°C; skim 1: 30 V; capillary exit: 75 V; capillary voltage: 1800 V; for each precursor ion two averages were taken; ion current control: on; trap drive: 85; smart target: 500,000; MS scan range: 400–1600; maximum accumulation time: 150 ms; ultra scan: on averages: 1; fragmentation amplitude: 1.25 V; MS/MS: number of parents: 5; SmartFrag: on, 30–200%; spectra were actively excluded for fragmentation after two recorded spectra for 1 min to allow the detection of less abundant co-eluting compounds; exclude +1: on, MS/MS scan range: 100–2000; prefer +2: on; ion current control target: 500,000; ultra scan: on. 

### Database Search [7]

Database searches were performed against the IPI human database (http://www.ebi.ac.uk/IPI) with the Spectrum Mill Proteomics Workbench Rev A.03.03.078 software (Agilent Technologies). Peak lists were created with the Spectrum Mill Data Extractor program under the following conditions: scans with the same precursor ±1.4 m/z were merged within a time frame of ±15 s. Charges up to a maximum of 5 were assigned to the precursor ion, and the ^12^C peak was determined by the Data Extractor. Precursor ions needed to have a minimum signal to noise value of 25. Two missed cleavages were allowed. Peptides were automatically identified by the Spectrum Mill software using IPI human database (version 3.43) for tryptic peptides with the restriction to *Homo sapiens*. A mass tolerance of ±2.5Da for the precursor ions and a tolerance of ±0.7Da for the fragment ions were used. A Spectrum Mill autovalidation was performed first in the protein details mode and then in the peptide mode. Minimum scores, minimum scored peak intensity (SPI), forward minus reversed score threshold, and rank 1 minus rank 2 score threshold for peptides were dependent on the assigned precursor charge [7]. 

All protein hits found in a distinct database search by Spectrum Mill are non-redundant. To eliminate redundancy, the Protein Summary Mode groups all proteins that have at least one common peptide, and only the highest scoring member of each protein group is shown and counted in the protein list.

### Gene Ontology (GO) analysis of serum differential proteome dataset [7]

We used the BiNGO plugin to find statistically over-represented GO categories in biologic data as a tool for enrichment analysis of serum differential proteome dataset between LN+ER/PR-Her2+ dataset and LN-ER/PR+Her2- dataset. For enrichment analysis, we needed a test dataset (which is the identified serum differential proteome) and a reference set of GO annotation for the complete human proteome. The analysis was performed using the “hyper geometric test”, and all GO terms that were significant with *P < 0.00001* (after correcting for multiple term testing by Benjamini and Hochberg false discovery rate corrections) were selected as over-represented.

### ELISA

Serum proteins were prepared using a lysis buffer containing protease inhibitor cocktail 8340 (Abcam, Cambridge, UK). The protein amount in the serum concentrates was measured using a Coomassie Protein Assay Kit (Pierce, Rockford, IL, USA). Levels of sCD14 in serum samples were measured using an ELISA kit (DuoSet, R&D Systems, Minneapolis, MN) according the manufacturer’s instructions.

### Statistical Analysis

Unpaired t-test and one-way ANOVA were used to analyze correlation between the groups. A stepwise model selection process was used to arrive at a parsimonious model. The logistic regression modeling was employed to describe the relationship between levels of serum sCD14 and the risk of breast cancer recurrence. Receiver operating characteristic (ROC) curves were plotted to evaluate the sensitivity and specificity of the biomarker measurements in predicting recurrence of breast cancer. A two-tailed *P* value less than 0.05 were considered significant. Continuous variables are expressed as mean with or without standard deviation unless otherwise indicated.

## Results

### Serum sCD14 was significantly different between LN-ER/PR+Her2- and LN+ER/PR-Her2+ status breast cancer groups

**Figure 1:**
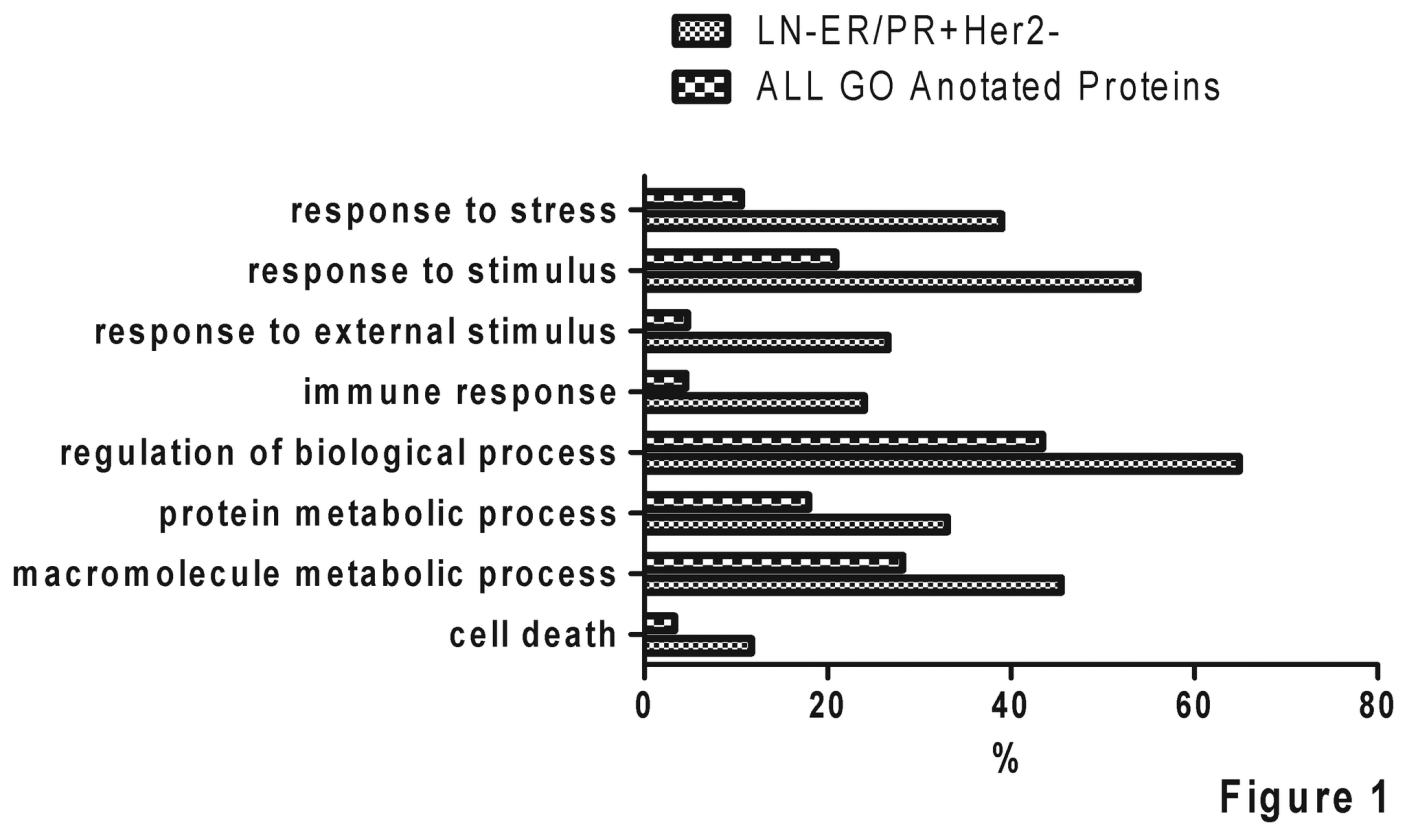
The comparative serum proteome analysis of breast cancer patients with different risk of recurrence. Serum proteome datasets of breast cancer patients with a lower risk of recurrence (LN-ER/PR+Her2-, n=50) or patients with a higher risk of recurrence (LN+ER/PR-Her2+, n=50) were obtained by using three different fractionation techniques (i.e. in-gel, 2DLC, or mRP) coupled with HPLC-CHIP-MS/MS. According to the criterion of differential protein as spectra counts ratio of LN-ER/PR+Her2- and LN+ER/PR-Her2+ datasets ≥10 or ≤0.1, the 70 significantly over-expressed proteins in LN-ER/PR+Her2- status were obtained. Following GO analysis, those 70 differential proteins were showed significantly over-represented in terms “response to biotic stimulus”, “response to endogenous stimulus”, “response to external stimulus”, “response to stimulus”, and “response to stress” (p<0.0001).

A total of 263 non-redundant high abundance proteins in serum of breast invasive ductal carcinoma patients with high confidence were identified by the technique of 2D-LC coupled with HPLC-CHIP-MS/MS (Data S1). To analyze serum differential proteins between patients with LN-ER/PR+Her2- status and LN+ER/PR-Her2+ status, we arbitrarily set the criterion of differential protein as spectra counts ratio of LN-ER/PR+Her2- and LN+ER/PR-Her2+ datasets ≥10 or ≤0.1. Comparison of these two datasets, 118 differential proteins were obtained, among which 70 were from the dataset of LN-ER/PR+Her2- status and 48 from the dataset of LN+ER/PR-Her2+ status (Data S2). Following GO analysis, the 70 significantly over-expressed proteins in LN-ER/PR+Her2- status were significantly over-represented in terms “response to biotic stimulus”, “response to external stimulus” and “response to stress” (Figure 1, *P < 0.00001*). It’s indicated that immune status of breast cancer patients was an important factor for tumor recurrence. Among the 70 proteins, there were 10 immune-related proteins (Table 2). Interestingly, CD14 was the most abundant serum protein among the 10 immune-related proteins, which was detected with >3 unique peptides and >100 spectrum counts. Therefore, CD14, an important molecule of innate immunity, was selected for further validation in following study.

**Table 2:** The differential immune-related proteins identified in serum of LN-ER/PR+Her2- status group.

**Uniprot ID**		**Protein Definition**
P54652.1	Heat shock-related 70 kDa protein 2	
P49959.3	cDNA FLJ38069 fis, clone CTONG2015434, highly similar to DOUBLE-STRAND BREAK REPAIR PROTEIN MRE11A	
P08571.2	Monocyte differentiation antigen CD14	
P67937.3	Tropomyosin alpha-4 chain	
P78536.1	Disintegrin and metalloproteinase domain-containing protein 17	
P51587.2	Breast cancer type 2 susceptibility protein	
O75636.2	Ficolin-3	
P01009.3	Alpha-1-antitrypsin	
Q86W56.1	Poly(ADP-ribose) glycohydrolase	
Q8TC71.1	Mitochondria-eating protein	

Serum sCD14 was significantly lower in LN+ER/PR-Her2+ status breast cancer patients than in LN-ER/PR+Her2- status breast cancer patients

**Figure 2:**
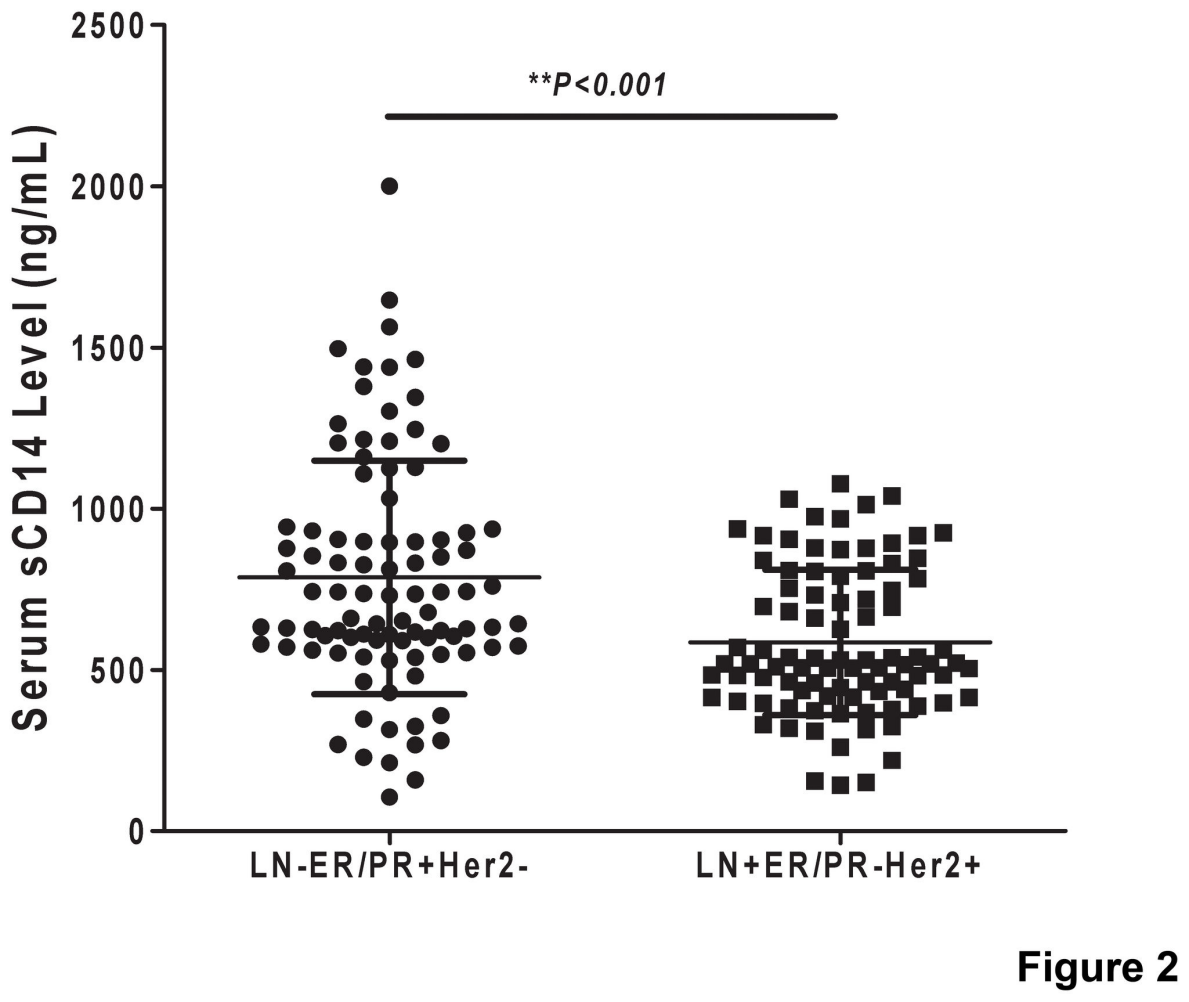
Determination of serum sCD14 concentrations in patients of LN-ER/PR+Her2- and LN+ER/PR- Her2+ phenotype breast cancer by ELISA. A significantly higher level of serum sCD14 was observed in patients with LN-ER/PR+Her2- status (n=93) than in that with LN+ER/PR-Her2+ status (n=90) (p<0.0001).

To confirm the preliminary proteomics result, serum sCD14 of 183 subjects (LN-ER/PR+Her2-, n=93; LN+ER/PR-Her2+, n=90) were examined via ELISA (Data S2). The result showed that the average level of serum sCD14 was significantly higher in LN-ER/PR+Her2- status group than that in LN+ER/PR-Her2+ status group (Figure 2, *p<0.001*). The median difference in serum sCD14 levels between the LN-ER/PR+Her2- status and LN+ER/PR-Her2+ status groups was 201.7±44.73 ng/ml.

### Serum sCD14 was significantly lower in the 3 years recurrent patients than in the recurrent-free patients

**Figure 3:**
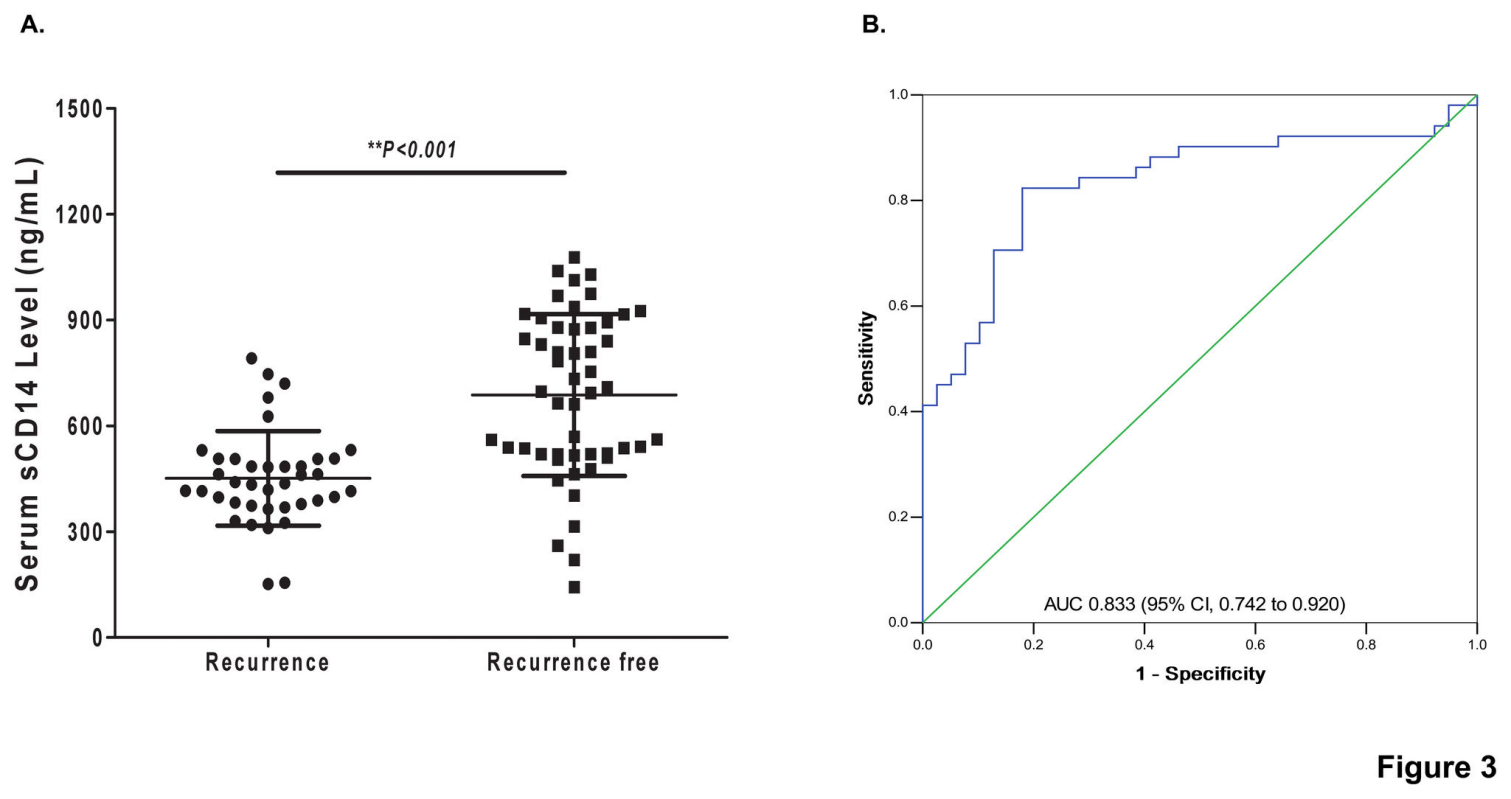
Pre-validation of serum sCD14 as a biomarker for predicting the recurrence of breast invasive ductal carcinoma with LN+ER/PR-Her2+. (A) Comparison of the level of serum sCD14 in relapse and relapse-free patients of breast cancer with LN+ER/PR-Her2+ status. The level of serum sCD14 was significantly lower in the patients with recurrence than those without recurrence (p<0.001). (B) The receiver operating characteristics (ROC) curve of serum sCD14. The AUC was 0.833 (95% CI, 0.742 to 0.920).

To know whether or not the serum sCD14 could act as a biomarker for prediction of the LN+ER/PR-Her2+ status breast cancer recurrence, a 3-years follow-up study on the 90 LN+ER/PR-Her2+ status breast cancer patients was conducted. The follow-up data showed that, among 90 LN+ER/PR-Her2+ status breast cancer patients, 39 were later found to have recurrence in 3 years after primary surgery; the cancer relapse rate was 43.3%. In cancer recurrent group, the median level of serum sCD14 was 451.3 ng/mL (interquartile range from 377.99 to 506.33 ng/mL), while it was 687.5 ng/mL (interquartile range from 518.99 to 894.03 ng/mL) for cancer recurrence-free group (Figure 3A, *p<0.001*). However, there was no significant variation of serum sCD14 among the different groups classified according to age (≤35; 36-55; >55), the stage of tumor (I; IIa; IIb; IIIa), the size of tumor (≤2cm; 2-5cm; >5cm), the intensity of Her2-positive (Her2+; Her2++; Her2+++), or the lymph node (1-3; 4-9; ≥10) (Figure S1). A logistic regression model was further used to estimate the relationship between serum sCD14 and the risk of breast cancer recurrence. The result showed that the correlation between serum sCD14 levels and the risk of breast cancer recurrence was statistically significant (Table 3, coefficient factor was -0.672, *p<0.001*). However, other clinical observation and traditional tumor biomarkers were not significantly associated with the risk of LN+ER/PR-Her2+ status breast cancer recurrence (Table 3).

**Table 3:** Relationship between biomarkers, clinical characteristics and the risk of LN+ER/PR-Her2+ status breast cancer.

Variable	Coefficient	SE	*P*
Level of sCD14	-0.672	0.158	<0.001
Age	0.09	0.054	0.114
Stage Classification	0.04	0.649	0.949
Tumor Size	0.43	0.416	0.297
Number of Lymph Node Metastasis	0.06	0.085	0.468
Content of Her2 %	0.36	0.770	0.644
Content of PCNA %	0.03	0.026	0.269
Content of p53 %	-0.03	0.029	0.254

NOTE. A logistic regression model was used to estimate the odds of breast cancer recurrence, adjusted for all of the variables listed in the table.

To access the value of the serum sCD14 as prediction biomarker, ROC curve was then constructed for the serum sCD14. The area under curve (AUC) of the ROC curve for sCD14 was 0.833 (Figure 3B, 95% CI, and 0.742 to 0.920). The AUC for the PCNA ROC curve was 0.556 (95% CI, 0.433 to 0.68), and that for Her2 was 0.525 (95%CI, 0.405 to 0.645), for P53 was 0.396 (95%CI, 0.237 to 0.555), for LN was 0.615 (95%CI, 0.494 to 0.735), for the stage of tumor was 0.669 (95%CI, 0.556 to 0.783), and for tumor size was 0.612 (95%CI, 0.496 to 0.729) (Figure S2). When a concentration of 508.84 ng/mL of serum sCD14 was set up as the threshold, the positive predictive value (PPV) was 82.4% and the negative predictive value (NPV) was 82.1%.

## Discussion

Generally, breast cancers with LN+ER/PR-Her2+ status phonotype are practically considered at high risk for recurrence [2,3]. However, no report on the accurately recurrent rate of LN+ER/PR-Her2+ status breast cancer has been found. Our current limited data showed that the recurrent rate of LN+ER/PR-Her2+ status breast cancer was about 43.3% and the recurrent rate of LN-ER/PR+Her2- status breast cancer was about 12.9% in 3 years (Table 1). Unfortunately, current clinical observation and traditional tumor biomarkers were not significantly associated with the risk of those breast cancer recurrences (Table 3 and Figure S2). There is not even any effective biomarker for specifically predicting of those phenotype cancers up-to-now. Herein, we try to identify the breast cancer recurrence-related proteins through using the comparative proteomics to analyze the differential proteins between the breast invasive ductal carcinoma with a higher risk of recurrence (LN+ER/PR-Her2+) and those with a lower risk of recurrence (LN-ER/PR+Her2-). The level of serum sCD14 was pre-validated as a biomarker for predicting recurrence of breast invasive ductal carcinoma with LN+ER/PR-Her2+ status in a single center retrospective study. In LN+ER/PR-Her2+ status group, the level of serum sCD14 was significantly lower in patients with recurrence in 3 years than those without recurrence (Figure 3A), and was significantly correlated to the risk of 3-year cancer recurrence as well. The corresponding AUC was 0.833 (Figure 3B, 95% CI, and 0.742 to 0.920). This led us to propose serum sCD14 as a novel potential biomarker for predicting the recurrence of LN+ER/PR-Her2+ status breast cancer.

**Figure 4:**
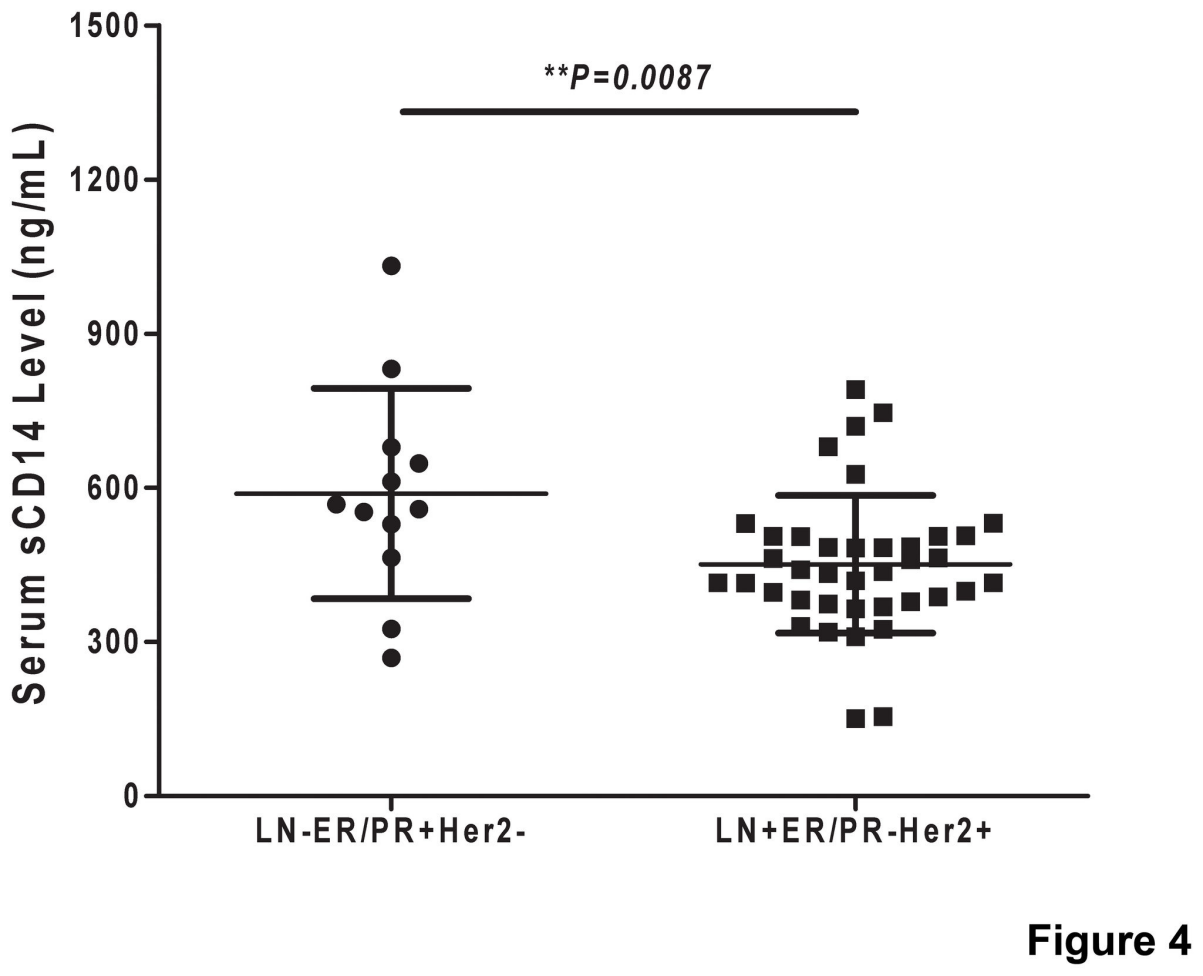
Comparison of serum sCD14 levels between the recurrent patients of breast cancer with LN-ER/PR+Her2- status and those with LN+ER/PR-Her2+ phenotype. The level of serum sCD14 was significantly higher in LN-ER/PR+Her2- status relapse patients than in LN+ER/PR-Her2+ status relapse patients (p<0.001).

Another important finding is that serum sCD14 was shown to be probably valuable for predicting the recurrence of breast cancer with LN-ER/PR+Her2- status after the analysis of those 12 relapsed patients in 3 years in enrolled 93LN-ER/PR+Her2- breast cancer patients. In this group, the level of serum sCD14 was significantly lower in patients with recurrence in 3 years (589.1±59.12 ng/mL, n=12) than those without recurrence (Figure S3a, 828.5±39.71 ng/mL, n=81, *p=0.262*), and the AUC was 0.731 (Figure S3b, 95% CI, 0.591 to 0.872). However, the sCD14 threshold is different for predicting the recurrence of LN-ER/PR+Her2- status and LN+ER/PR-Her2+ status breast cancer. Our result showed that the level of serum sCD14 was significantly higher in recurrent patients with LN-ER/PR+Her2- status (589.1±59.12 ng/mL, n=12) than those with LN+ER/PR-Her2+ status (Figure 4, 451.3±21.42 ng/mL, n=81, *p=0.0087*). The cutoff value of sCD14 was 704.79 ng/mL with 55.6% sensitivity and 83.3% specificity in LN-ER/PR+Her2- status group, whereas, the cutoff value reduced to 508.84 ng/mL with 82.4% sensitivity and 82.1% specificity in LN+ER/PR-Her2+ status group. It indicates that serum sCD14 thresholds could be different in cohort with different tumor status.

It is well-known that the immune status of patient is one of the important factors to influence cancer prognosis [8], while only a few potential biomarkers related to immune system for cancer prognosis were identified [8,9,10,11]. Our study confirmed that the differential serum proteins between LN-ER/PR+Her2- and LN+ER/PR-Her2+ status breast cancers were significantly related to immune function, particularly related to serum sCD14. CD14 is an important component of the innate immune Toll-like receptor system and gram-negative and gram-positive bacterial pattern recognition [12]. The soluble form of CD14, that lacks the glycosylphosphatidyl inositol tail, was abundant in serum [13] as well as in urine [7]. A few studies reported that the serum level of sCD14 was higher in patients with cancer than in patients with benign disease or healthy people [14,15,16], and therefore sCD14 has been considered to possibly play a part in immune tolerance [17,18] and in cancer development [16]. Here, we for the first time demonstrated that the breast cancer patients with a lower serum sCD14 level were at significantly higher risk of recurrence than those with higher serum sCD14 level. However, the underlying mechanism of the relationship between breast cancer recurrence and the serum sCD14 level has yet to be clarified.

## Conclusion

In summary, we for the first time showed that serum sCD14 could be served as a biomarker for prediction of LN+ER/PR-Her2+ status breast cancer recurrence. Multiple clinical center validation is required prior to any application in clinical settings.

## Supporting Information

Figure S1 **Serum sCD14 levels in different age, tumor stage classification, Her2 content, LN+, tumor sizes.** The levels of serum sCD14 were not significantly different among the different groups classified according to age(≤35; 36-55; >55), the stage of tumor (I; IIa; IIb; IIIa), the size of tumor (≤2cm; 2-5cm; >5cm), the intensity of Her2-positive (Her2+; Her2++; Her2+++), or the lymph node (1-3; 4-9; ≥10) in LN+ER/PR-Her2+ phenotype subjects (P>0.05).
(TIF)

Figure S2 **The ROC curves of clinical factors and biomarkers.** The AUC for the PCNA ROC curve was 0.556 (95% CI, 0.433 to 0.68), and that for Her2 was 0.525 (95%CI, 0.405 to 0.645), for P53 was 0.396 (95%CI, 0.237 to 0.555), for LN was 0.615 (95%CI, 0.494 to 0.735), for the stage of tumor was 0.669 (95%CI, 0.556 to 0.783), and for tumor size was 0.612 (95%CI, 0.496 to 0.729).
(TIF)

Figure S3 **Pre-validation of Serum sCD14 as a biomarker for predicting the recurrence of breast invasive ductal carcinoma with LN-ER/PR+Her2** **-**. (A) Comparison of the level of serum sCD14 in relapse and relapse-free patients of Breast Cancer with LN-ER/PR+Her2- phenotype. The level of serum sCD14 was significantly lower in the patients with recurrence than those without (*P<0.001*). (B) The receiver operating characteristics (ROC) curve of serum sCD14. The AUC was 0.788 (95% CI, 0.593 to 0.983).
(TIF)

Data S1 **Serum proteome datasets of breast cancer patients with LN-ER/PR+Her2- status and patients with LN+ER/PR-Her2+ status.**
(XLS)

Data S2 **ELISA results of serum sCD14 concentration in the 183 breast cancer patients.**
(XLS)
