# Supplementary figures and images for: Serum Soluble CD14 Is a Potential Prognostic Indicator of Recurrence of Human Breast Invasive Ductal Carcinoma with Her2-Enriched Subtype
Source: PLoS One. 2013 Sep 25;8(9):e75366. doi: 10.1371/journal.pone.0075366 (PMC3783397; doi:10.1371/journal.pone.0075366)

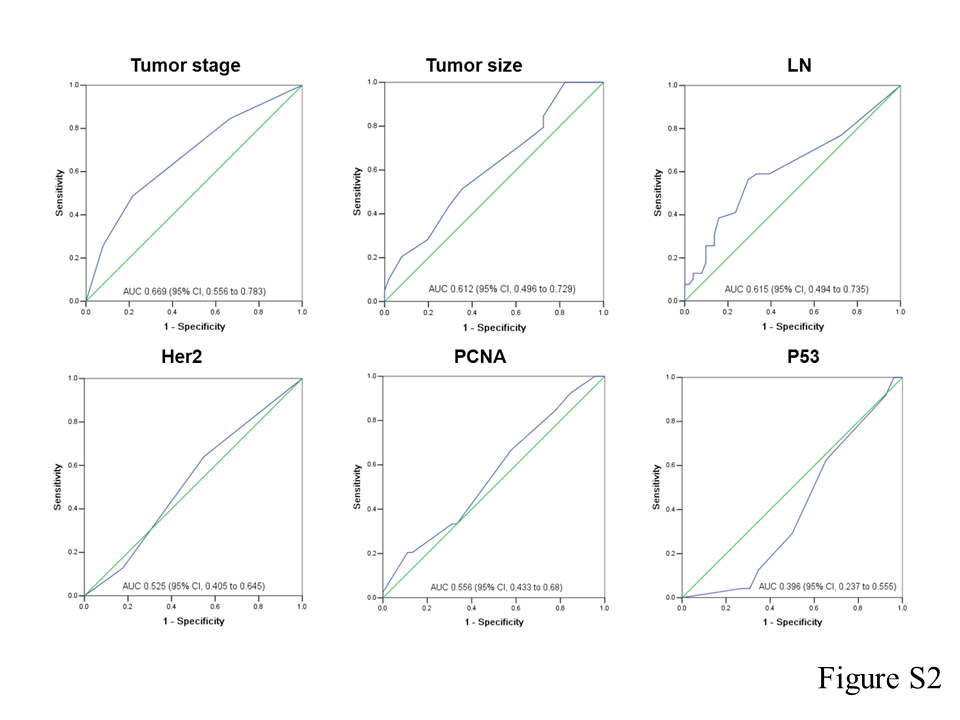

Supplement: Figure S2 — The ROC curves of clinical factors and biomarkers. The AUC for the PCNA ROC curve was 0.556 (95% CI, 0.433 to 0.68), and that for Her2 was 0.525 (95%CI, 0.405 to 0.645), for P53 was 0.396 (95%CI, 0.237 to 0.555), for LN was 0.615 (95%CI, 0.494 to 0.735), for the stage of tumor was 0.669 (95%CI, 0.556 to 0.783), and for tumor size was 0.612 (95%CI, 0.496 to 0.729). (TIF) [file pone.0075366.s002.tif]

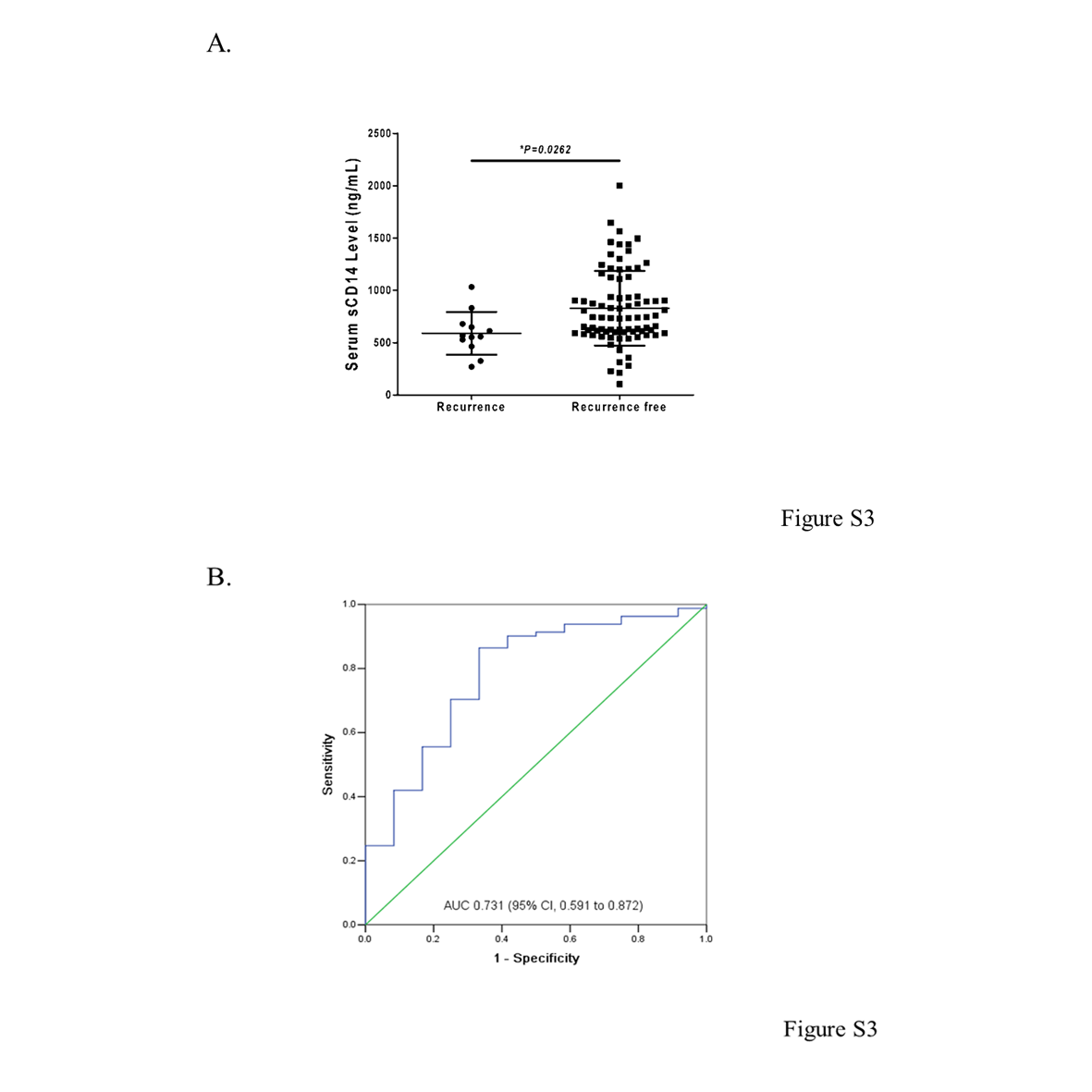

Supplement: Figure S3 — Pre-validation of Serum sCD14 as a biomarker for predicting the recurrence of breast invasive ductal carcinoma with LN-ER/PR+Her2 -. (A) Comparison of the level of serum sCD14 in relapse and relapse-free patients of Breast Cancer with LN-ER/PR+Her2- phenotype. The level of serum sCD14 was significantly lower in the patients with recurrence than those without (P<0.001). (B) The receiver operating characteristics (ROC) curve of serum sCD14. The AUC was 0.788 (95% CI, 0.593 to 0.983). (TIF) [file pone.0075366.s003.tif]
